# Interaction of *Cyprinus carpio* Linnaeus with the biofilm-forming *Aeromonas hydrophila*

**DOI:** 10.14202/vetworld.2022.2458-2465

**Published:** 2022-10-22

**Authors:** Ekaterina Lenchenko, Svyatoslav Lenchenko, Nadezhda Sachivkina, Olga Kuznetsova, Alfia Ibragimova

**Affiliations:** 1 Department of Veterinary Medicine, Moscow State University of Food Production, 125080, Moscow, Russia; 2 All-Russian Research Institute of Integrated Fish Farming – a branch of the Federal State Budgetary Scientific Institution “Federal Research Center for Livestock – VIZh named after Academician L.K. Ernst” (VNIIR – a branch of the LK Ernst Federal State Budgetary Scientific Institution FITS VIZH), 142460, Moscow region, Noginsky District, Pos. Them. Vorovskogo, Russia; 3 Department of Microbiology and Virology, Institute of Medicine, Peoples’ Friendship University of Russia (RUDN University), 117198 Moscow, Russia; 4 Department of Biochemistry, Institute of Medicine, Peoples’ Friendship University of Russia (RUDN University), 117198 Moscow, Russia; 5 Department of General Pharmaceutical and Biomedical Technologies, Institute of Medicine, Peoples’ Friendship University of Russia (RUDN University), 117198 Moscow, Russia; 6 Department of Foreign Languages, Institute of Medicine, Peoples’ Friendship University of Russia (RUDN University), 117198 Moscow, Russia

**Keywords:** adhesion, *Aeromonas hydrophila*, biofilm, *Cyprinus carpio* Linnaeus, optical density

## Abstract

**Background and Aim:**

The resistance of susceptible fish populations and the adaptive potential of heterogeneous biofilms, which cause multiple antibacterial resistance and long-term persistence of microorganisms, mediate the development and outcome of the infectious process. The study of the fish immunological parameters in interaction with biofilm-forming bacteria is of practical importance for assessing the stability of the homeostasis of the fish. This study aimed to determine the immunobiological parameters of *Cyprinus carpio* Linnaeus when interacting with biofilm-forming bacteria *Aeromonas hydrophila*.

**Materials and Methods:**

Clinically healthy fish *C. carpio* L. (Linnaeus, 1758) of both sexes, aged 4 years, and weighing 1.0–1.5 kg (n = 10), were used in this study. The fish were taken from the pond of the VNIIR experimental base in the period of 2020–2022. The standard method was employed to determine the phagocytic activity of blood cells, the total redox activity of neutrophils, and the bactericidal activity of blood serum.

**Results:**

After 24–48 h of cultivation in nutrient broth, the implementation of the processes of intercellular communication of bacteria had common patterns of formation of the heterogeneous structure of biofilms. Moreover, analyzing the optical density indices (density, D), it was observed that *A. hydrophila* was a strong producer of biofilms, as the optical density of the sample (density of sample, Ds) exceeded the optical density of the control (density of control, Dc) by more than 4 times (D = 0.464 ± 0.07). The ratio of the average number of microorganisms attached to the surface of one erythrocyte (average adhesion index) and the percentage (%) of erythrocytes having bacteria on their surface (adhesion coefficient [AC]) was 14.05 ± 0.72, and the adhesion index, AI was ≥4.00, indicating *A. hydrophila* to be highly adhesive. In addition, the AC of erythrocytes having bacteria on the surface was 14.05% ± 0.72%. A direct correlation was established (R^2^ = 0.94) between the AC (14.05% ± 0.11%–13.29% ± 0.08%) and the phagocytic index (11.3% ± 0.29%–32.0% ± 0.8%). The indicators of spontaneous nitro blue tetrazolium were 103.20 ± 11.70 when estimating the total redox activity of neutrophils. The optical density increased to 182.10 ± 21.12 with the addition of 20.0 mL of *A. hydrophila* bacteria (1 billion/mL) and the activity of neutrophils also increased.

**Conclusion:**

Among the markers of homeostasis stability, immunological indicators most fully reflect the mechanisms of initiation, development, and outcome of the infectious process mediated by the interaction of adhesive molecules of multicellular eukaryotes and adhesives of infectious disease pathogens. The research will contribute to further understanding the potential mechanism of quorum-sensing molecules and the search for new anti-adhesive drugs that reduce the formation of biofilms.

## Introduction

The rapid growth of anthropogenic loads and water body pollution contributes to a decrease in the indicators of natural resistance of the organisms of hydrobionts [1–5]. The immunosuppressive effect of antigens determines the risk of developing infectious pathology, and fish mortality reaches 80.0–100.0% [6]. A wide distribution of virulent *Aeromonas* spp., producing hemolysins (23.6%), enterotoxins (12.8%), and cytotoxic (36.7%), has been established [7]. *Aeromonas* spp. isolates from mullet and freshwater samples produced cytotoxic enterotoxin and displayed resistance to b-lactams and sulfonamides (100.0%), oxytetracycline (90.0%), and streptomycin (62.22%) [8].

The development and outcome of the infectious process are mediated both by the resistance of susceptible animal species and the adaptive potential of heterogeneous biofilms, which determine the phenotypic plasticity of the long-term persistence of microorganisms [9, 10]. Heteromorphic population growth contributes to the expansion of the adaptive potential of bacteria, which determines the virulence and protection of microbial biofilms from the immune response, as well as from the effects of chemotherapeutic drugs and disinfectants [11–13].

Furthermore, it is necessary to study the immunobiological parameters of fish blood in interaction with bacteria forming biofilms, which determined the relevance of the research to develop effective methods for assessing the natural resistance of hydrobionts. This study aimed to investigate the immunobiological parameters of *Cyprinus carpio* L. in interaction with the biofilm-forming bacteria *Aeromonas hydrophila*.

## Materials and Methods

### Ethical approval

The work was carried out complying with the requirements of international agreements “Directive 2010/63/EU of the European Parliament and the Council of the European Union,” dated September 22, 2010, on the protection of animals used for scientific purposes. All experiments were performed in accordance with the Guide for the Care and Use of Laboratory Animals [14] and approved by the Peoples’ Friendship University of Russia (RUDN University) ethical committee (EC1/201, February 10, 2022).

### Study period and location

The study was conducted from September 2020 to February 2022 in the Laboratory of All-Russian Research Institute of Integrated Fish Farming – a branch of the Federal State Budgetary Scientific Institution “Federal Research Center for Livestock – VIZh named after Academician L.K. Ernst”, Moscow region, Russia.

### Experimental animals

The studies were conducted on clinically healthy fish, *C. carpio* L. (Linnaeus, 1758), caught from the pond of the VNIIR experimental base in the period of 2020–2022. The fish were of both sexes, aged 4 years (4 years old), and weighed 1.0–1.5 kg (n = 10).

### Bacterial strain

The reference strain of *A. hydrophila* ATCC 22492 bacteria from the collection of the State Research Institute for Standardization and Control of Medical Biological Preparations, named after L.A. Tarasevich (Moscow) was used in the experiments [15].

### Nutrient media

Microorganisms were cultured at 22°C ± 1°C for 24–48 h on Difco Bacto agar (Difco, USA), nutrient broth (HiMedia, India), and blood agar base (BioMerieux, France) media.

### Morphometric and densitometric indicators of bacterial biofilms

The morphometric and densitometric indicators of bacterial biofilms were studied using flat-bottomed, sterile, 12-well culture plates with lids with the treated surface for monolayer cell cultures. The volume of the wells was 6.8 mL (Medpolymer, Russia), and small glasses for microbiological studies were placed at the bottom of the holes (18.0 × 18.0 mm) (Corning Inc., USA). Later, 3.0 mL of nutrient broth (HiMedia, India) and 1.0 mL of a bacterial suspension of *A. hydrophila* culture in concentration 0.5 units (McFarland) were placed together in the wells. Microorganisms were cultured for 24 and 48 h at 22°C ± 1°C. The plates were mixed at 2000 rpm for 10 min using a vortex shaker (MixMate) (“Eppendorf,” Germany) before and after cultivation. Biofilm preparations on the glass surface were fixed with a mixture of alcohol and ether (1:1) for 10 min. For optical microscopy, the preparations were stained with an aqueous solution of gentian violet (dilution 1:2000) for Gram staining (BioVitrum, Russia) [9].

Morphometric studies were carried out with a representative sample of a reliable frequency of occurrence ≥90.0% of the field of view of the optical microscope (Optika M B-353LD1) (“Optika M,”), equipped with a digital imaging module for the display (HP Z Display) (“Optika M,” Italy). Furthermore, for scanning electron microscopy (Hitachi TM4000Plus) (“Hitachi,” Japan) analysis, the samples were sputtered with gold ions (Q150T ES) (“Quorum Technologies,” Great Britain) [11, 12].

The optical density of biofilms was determined by the degree of binding of crystal violet. That is, for weak biofilm producers, the optical density of the sample or the culture of microorganisms (density of the sample, Ds) is greater than the optical density of the control (OD_C_) (density of the control, Dc) and is < 2 times (D ≤ 0.197); the Ds exceeds the OD_C_ by 2–4 times (D = 0.279–0.571) for moderate biofilm producers; and the Ds exceeds the OD_C_ by more than 4 times (D = 0.699–1.510) for strong biofilm producers [13].

### Adhesive properties of bacteria and phagocytic activity of blood cells

The blood of fish aged for 5–10 min in aerated water (O_2_ ≥ 5.0 mg/L) was taken from the tail vein for the study. The instruments were pretreated with 3.8% aqueous sodium citrate solution (Famar Health Care Services, France) [16].

Further, the adhesive properties of bacteria and phagocytosis of fish blood cells were analyzed. Briefly, 2.0 mL of blood and 0.1 mL of 2.0% sterile sodium citrate solution were injected into 5.0 mL test tubes (Eppendorf). Later, 0.5 mL of a bacterial suspension of *A. hydrophila*, 1 billion/mL pre-inactivated at 60°C for 30 min, was introduced. The studied samples were homogenized using the RM 100 Touch mixer (Lamy Rheology, France) and cultured at 22°C ± 1°C for 15 and 120 min [17].

Blood smears were fixed with an alcohol-ether mixture (1:1) for 10 min and stained with a dye according to May-Grunwald (BioVitrum). Taking into account at least 50 erythrocytes, the following indicators were determined: Average adhesion index (AAI), the average number of microorganisms attached to the surface of one erythrocyte; adhesion coefficient (AC), the percentage of erythrocytes having bacteria on their surface; and adhesion index (AI), the ratio of AAI and AC indicators. Depending on the values of the adhesion index of microorganisms, AI = 1.00–1.75 bacteria were considered non-adhesive; AI = 1.76–2.49 were low adhesive; AI = 2.50–3.99 were medium adhesive; and AI ≥ 4.00 were high adhesive. Furthermore, to account for the phagocytic activity of blood cells, the following indicators were determined: Phagocytic number, the ratio of the number of microorganisms absorbed by each leukocyte and the number of leukocytes absorbed by microorganisms; and phagocytic index, the ratio of the number of leukocytes absorbed by microorganisms and the total number of leukocytes [18].

The total redox activity of leukocytes under the action of reactive oxygen species was determined by the reduction of the nitro blue tetrazolium (NBT) test. Briefly, 90.0 μL of blood and 10.0 μL of 2.0% sterile sodium citrate solution were injected into wells with a volume of 400.0 μL of 96-well microtiter plates (Nalge Nunc, UK) and incubated at 25°C ± 1°C for 120 min. To take into account the spontaneous NBT reduction and OD_C_ indicators, a 100.0 μL solution of NBT reagent (1.0 mg/mL) (Sigma-Aldrich, USA) was introduced into the wells of the plate. The NBT-induced OD_S_ indicators was taken into account when adding 100.0 μL of NBT reagent solution and 20.0 μL of *A. hydrophila* (1 billion/mL). The studied samples were cultured at 22°C ± 1°C for 60 min, and the optical density of sample 1, OD_S1_, was determined at a wavelength of 630 nm (“Immunochem-2100 HTI,” USA) [19].

### Bactericidal activity of blood serum

The bactericidal activity of blood serum was also determined. Briefly, 2.5 mL of nutrient broth, 0.1 mL of blood serum, 0.1 mL of 2.0% sterile sodium citrate solution, and 0.2 mL of bacterial suspension (1 billion/mL) of *A. hydrophila* were injected into 10.0 mL test tubes, and the OD_S_ was recorded. Observing the principles of analogues, 2.5 mL of nutrient broth, 0.5 mL of 0.85% NaCl solution, and 0.2 mL of bacterial suspension (1 billion/mL) of *A. hydrophila* were added to the test tubes, and later, the OD_C_ was determined. The studied samples were cultured at 22°C ± 1°C for 180 min and the OD was determined as OD_S1_, OD_C1_. To determine the bactericidal activity of blood serum (%), the ratio of optical density indicators was taken into account: 100-(OD_c1_-OD_c_/OD_s1_-OD_s_) [17].

The ODs of the studied samples was determined at a wavelength of 490 nm using a microplate photometric analyzer (Immunochem-2100 [“HTI,” USA]).

### Statistical analysis

Experimental data were processed using descriptive and logical statistics. The average values and standard deviations of optical densities, adhesive properties of bacteria, and phagocytic activity of hemocytes were calculated using Microsoft Excel. The difference between the mean samples and the control values was determined using the Student’s t-test, and the statistical significance of the differences was established at the level of p ≤ 0.05.

## Results

**Figure-1:**
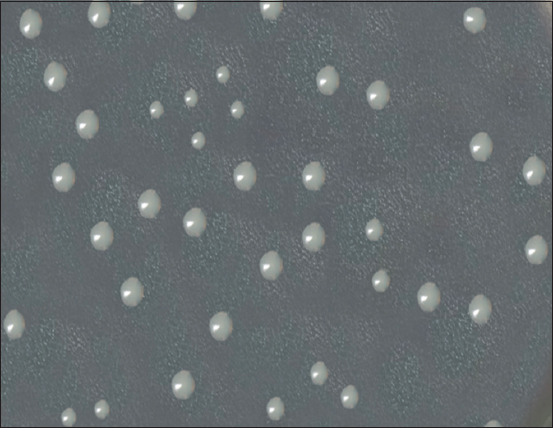
Morphology of *Aeromonas hydrophila* colonies, “Difco Bacto agar,” 22 ± 1°C, 24 h.

*Aeromonas hydrophila* bacteria are Gram-negative mobile rods that do not form spores and capsules, facultative aerobes, oxidase and catalase positive, fermented glucose and maltose to form acid, and reduced nitrates. Furthermore, on the Bacto agar medium, the bacteria formed S-forms of colonies after 24 h of cultivation. Figure-1 illustrates rounded, convex with smooth edges, and shiny, translucent colonies of microorganisms.

### Morphometric indicators of bacterial biofilms

**Figure-2:**
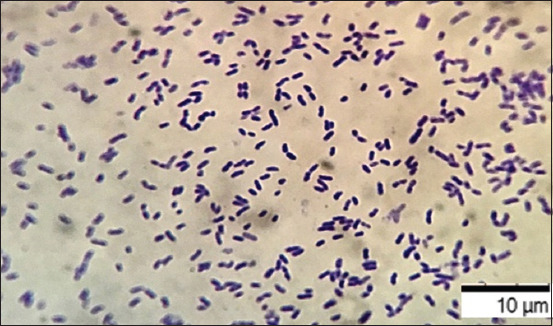
Culture of microorganisms *Aeromonas hydrophila* ATCC 22492, “Nutrient Broth,” 22 ± 1°C, 24 h: Rod-shaped bacteria united by an intercellular matrix. Gentian violet staining, optical microscopy, 900×, immersion (Optika M B-353LD1, Italy).

**Figure-3:**
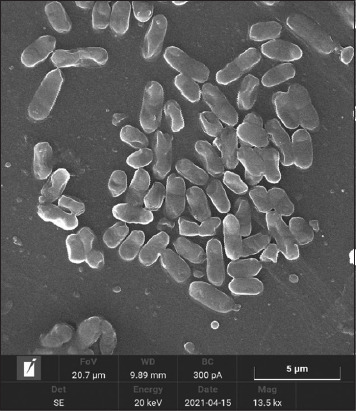
Culture of *Aeromonas hydrophila* ATCC 22492 microorganisms, “Nutrient Both” medium: 22 ± 1°C, 24 h: Densely arranged bacteria united by an exocellular matrix. SM × 3000 (“Hitachi TM 4000 Plus,” Japan).

After 24–48 h of cultivation in the nutrient broth medium, the implementation of the processes of intercellular communication of bacteria had common patterns of formation of the heterogeneous structure of biofilms. At the early stages of cultivation, adhesion was observed, that is, the attachment of planktonic cells to the surface of the substrate (Figures-2 and 3).

**Figure-4:**
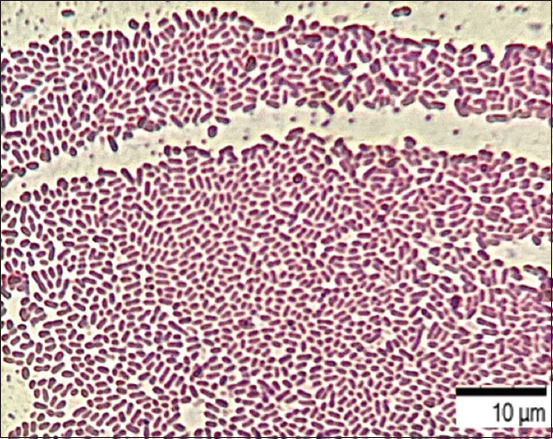
Culture of microorganisms *Aeromonas hydrophila* ATCC 22492, “Nutrient Broth,” 22 ± 1°C, 48 h: Rod-shaped bacteria of typical shape and size, united by an intercellular matrix. Gentian violet staining, optical microscopy, 900×, immersion (Optika M B-353LD1, Italy).

**Figure-5:**
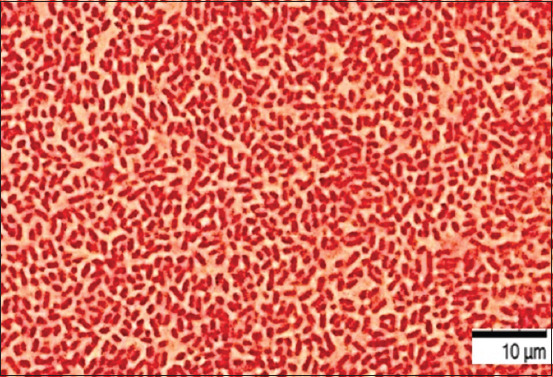
Culture of microorganisms *Aeromonas hydrophila* ATCC 22492, “Nutrient Broth,” 22 ± 1°C, 48 h: Gram-negative rod-shaped bacteria of typical shape and size, united by an intercellular matrix. Gram staining, optical microscopy, 900×, immersion (Optika M B-353LD1, Italy).

**Figure-6:**
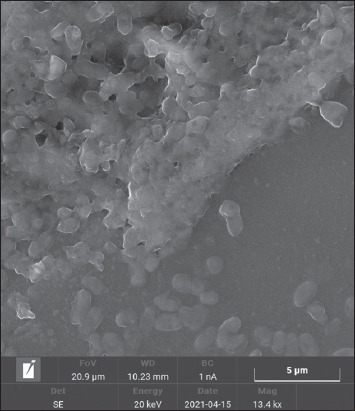
Culture of *Aeromonas hydrophila* ATCC 22492 microorganisms, “Nutrient Broth” medium: 22 ± 1°C, 48 h: A microcolony site of densely arranged bacteria united by an exocellular matrix. SM × 3000 (“Hitachi TM 4000 Plus,” Japan).

The final (irreversible) attachment of microorganisms occurred due to the release of cellular polymers that ensure the coagulation and strong fixation of cells to the substrate under study. Tightly packed microorganisms attached to the surface, held by the intercellular matrix, contributed to the attachment of subsequent cells, forming closed structures of various sizes, that is, a diffuse layer of cells. The formation of biofilm architectonics in the process of population development was ensured by the synthesis of a cellular matrix consisting of complex mobile gel structures. The presence of microorganisms attached to the substrate promotes the attachment of subsequent cells. The formation of secondary microcolonies was observed; some of the cells of the secondary microcolony were associated with the primary ones, and separated microcolonies separated by matrix voids were identified and characterized by the presence of cellular strands. Figures-4–6 show intercellular connections caused the immobilization of the bacterial population (Figures-4–6).

Mechanical stability was determined by cellular substances detected on the surface of biofilms in the form of polymer networks. Destructive processes of the intercellular matrix were revealed in some areas, including the dispersion of microorganisms that retained the ability to adhere to and forms new microcolonies. The formation of microcolonies in some areas was ensured by the process of coagulation – the formation of intercellular bonds and various densely packed cellular structures united by an intercellular matrix were revealed.

### Densitometric indicators of bacterial biofilms

Analyzing the OD indices, it was found that *A. hydrophila* were strong producers of biofilms as the ODs exceeded the OD_C_ by more than 4 times (D = 0.464 ± 0.07).

### Adhesive properties of bacteria

The phagocytic activity of eukaryotic cells and prokaryotic virulence factors both play a role in the initiation and outcome of the development of infectious pathology, so the adhesive qualities of bacteria that form biofilms were examined at the beginning of the study. After 30 min of interaction between the blood cells and bacteria, the AAI was 4.15 ± 0.28, and after 24 h of interaction, these indicators increased to 4.76 ± 0.20. The ratio of the average number of microorganisms attached to the surface of one erythrocyte (AAI) to % of erythrocytes having bacteria on their surface (AC) is 14.05 ± 0.72, and the adhesion index, AI is ≥4.00, indicating that *A. hydrophila* bacteria are highly adhesive. Furthermore, a direct correlation (R^2^ = 0.96) was established between the optical density of ODs biofilms (ODs = 0.464 ± 0.07) and the AAI = 4.76 ± 0.20).

### Phagocytic properties of fish blood cells

**Figure-7:**
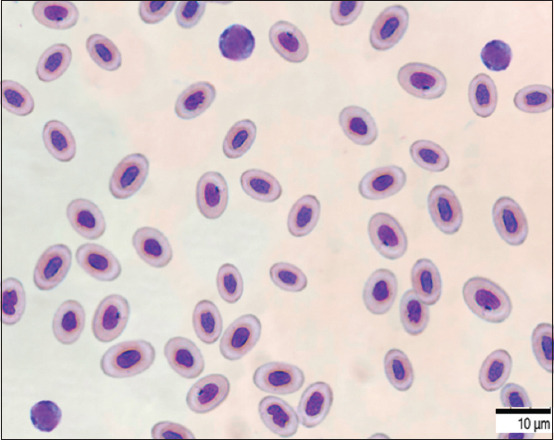
Blood smear of fish *Cyprinus carpio*, ϕ, 4 years old, 22 ± 1°C, 24 h. May-Grunwald staining, optical microscopy, 900×, immersion (Optika M B-353LD1, Italy).

**Figure-8:**
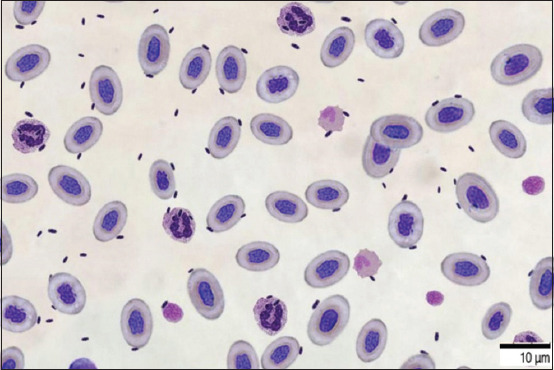
Blood smear of fish *Cyprinus carpio*, ϕ, 4-years-old, 22 ± 1°C, 24 h: Interaction of blood cells and *Aeromonas hydrophila* bacteria. May-Grunwald staining, optical microscopy, 900×, immersion (Optika M B-353LD1, Italy).

**Figure-9:**
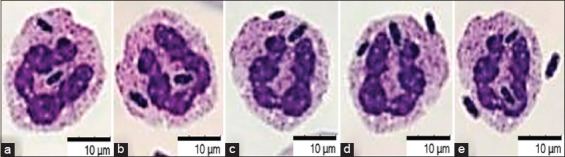
Fish blood smear *Cyprinus carpio*, ϕ, 4 years age, 22 ± 1°C, 24 h: (a-e) Interaction of blood cells and bacteria *Aeromonas hydrophila*. May-Grunwald staining, optical microscopy, 900×, immersion (Optika M B-353LD1, Italy).

The phagocytic activity of leukocytes of blood cells after 15 min of interaction and bacteria revealed that the phagocytic number was 2.7 ± 0.23, and the phagocytic index was 11.3 ± 0.29. Further, after 60 min of interaction, the phagocytic activity of fish blood cells increased, that is, the phagocytic number was 7.2 ± 0.02 and the phagocytic index was 32.0 ± 0.8 (Figures-7–9).

The AC of erythrocytes having bacteria on the surface is AC = 14.05% ± 0.72%. A direct correlation was established (R^2^ = 0.94) between the AC which was 14.05 ± 0.11–13.29% ± 0.08% and the phagocytic index (11.3% ± 0.29%–32.0% ± 0.8%). In addition, the total redox activity of neutrophils, which was a sign of spontaneous NBT was 103.20 ± 11.70. The activity of neutrophils increased, the ODs increased, and the amount reached 182.10 ± 21.12 after the addition of 20.0 L of *A. hydrophila* bacteria (1 billion/mL) NBT induced. The ratio of NBT induced to NBT spontaneous, or the simulation index was 1.77 ± 0.19 (Table-1).

**Table-1:** Results of determination of the total redox activity of fish neutrophils.

NBT test	OD		

	OD_c_	OD_s_	ODs_1_
NBT spontaneous	0.089±0.01	87.11±09.43	103.20±11.70
NBT induced	0.087±0.01	98.12±12.16	182.10±21.12
Stimulation index	1.00±0.01	1.13±0.16	1.77±0.19

NBT=Nitro blue tetrazolium, OD=Optical density, OD_C_=OD control, blood, and NBT, OD_S_, OD_S1_=OD test sample, blood, NBT, and culture of microorganisms, p ≤ 0.05

The bactericidal activity of fish blood serum was also determined. After 24 h, the changes in the studied samples containing *A. hydrophila* bacteria were observed. The optical density of the initial samples (ODs) was 0.423 ± 0.024. After 180 min of experimental studies, the optical density of the samples (ODs1) decreased to 0.088 ± 0.011. Bactericidal activity of blood serum (%), that is, the ratio of the difference (∆) of the indicated ODs indicators was 68.06 ± 1.14 (Table-2).

**Table-2:** Results of the determination of bactericidal activity of fish blood serum.

Recurrence	OD						

	OD_c_	OD_c1_	∆ (OD_c1_–OD_c_)	OD_s_	OD_s1_	∆ (OD_s_–OD_s1_)	∆ (OD_c1_–OD_c_)/∆ (OD_s_–OD_s1_), %
1	0,110	0,310	0,200	0,400	0,111	0,289	69,20
2	0,075	0,322	0,250	0,447	0,077	0,370	67,57
3	0,087	0,315	0,228	0,423	0,088	0,335	68,06

OD=Optical density; OD_C_, OD_C1_=Control, blood serum, and NaCl, OD_S_, OD_S1_=Experience, blood serum, and culture of microorganisms, ∆ (ODc–ODc1), ∆ (ODs–ODs1)=Difference between optical density indicators, ∆ (ODc1–ODc)/∆ (ODs– ODs1) (%)=Bactericidal activity of blood serum

## Discussion

Among the markers of homeostasis stability, immunological indicators most fully reflect the mechanisms of initiation, development, and outcome of the infectious process mediated by the interaction of adhesive molecules of multicellular eukaryotes and adhesives of infectious disease pathogens [9, 12, 19]. The immunological parameters of fish depend on the season of the year, so the greatest phagocytic activity and the activity of the complement of blood plasma were observed in autumn, decreased in summer and winter, and the least activity was detected in the spring period [20, 21]. In the process of immune reactions of vertebrates, including *C. carpio*, the stage of antigen presentation is crucial [12]. Antigen processing and presentation are a multi-stage process in which the antigen is absorbed by antigen-presenting cells and processed into peptide molecules, which are expressed on the cell surface by the peptide molecular complex and recognized by the T-cell receptor, activating specific immune responses of *C. carpio* [22]. With the development of pathology, along with the study of delayed hypersensitivity reactions, it is recommended to evaluate virulence factors and markers associated with the virulence plasmid of infectious disease pathogens [9, 10, 19]. Atomic force microscopy is recognized as a promising research method for assessing the adhesive properties of the plasmalemma of prokaryotic and eukaryotic cells; cytochemical and electron microscopic studies for studies of synapse destruction; and glycogen reduction in myeloid and lymphoid cells [23].

To block the synthesis or destruction of the intercellular matrix of biofilms during the development of superficial, deep, and systemic candidiasis, the fungistatic effect of farnesol is achieved at concentrations of 25, 50, and 100 microns [24–26]. Before this, the general patterns of intercellular communication in the formation of heterogeneous structures of microbial biofilms were established [27–29]. In the diet of fish, it is recommended to use prebiotics, probiotics, oligosaccharides, and tryptophan to increase the phagocytic activity of blood cells, the production of intracellular oxidative radicals, and the expression of intestinal cytokines and antioxidant genes [5, 18, 20]. In combination with increased sensitivity to chemotherapeutic and disinfecting drugs, the effect of reducing pathogenicity is achieved by reducing the formation of biofilms [30–32]. In addition, the probiotic *Pediococcus pentosaceus* to the diet at a dose of 1 × 10^8^ colony-forming unit/g allowed to increase (p < 0.05) in the activity of protease by 14.3% and lysozyme by 21.2%, the number of erythrocytes by 11.7%, and leukocytes by 8.22% [4]. Similarly, enriching the diet of *C. carpio* carp with pseudomonosis with extracts of *Euphorbia hirta* leaves led to a decrease in the number of *Pseudomonas fluorescens* microorganisms by 25.9%, and the concentration of hemoglobin increased −9.06 ± 0.103–10.67 ± 0.577 g/dL [33].

## Conclusion

The study of immunological parameters in interaction with biofilm-forming bacteria is of practical importance for assessing the stability of homeostasis of susceptible species. Among the markers of homeostasis stability, immunological indicators reflect the mechanisms of initiation, development, and outcome of the infectious process mediated by the interaction of adhesive molecules of multicellular eukaryotes and adhesives of infectious disease pathogens. We had proved that when *A. hydrophyla* bacteria were introduced, the phagocytic activity of fish blood cells increased, neutrophil activity increased, adhesion indices increased, and the bactericidal activity of blood serum increased. With the development of infectious pathology, along with the study of delayed hypersensitivity reactions, it is recommended to evaluate virulence factors and markers associated with the virulence plasmid of infectious disease pathogens. The research will contribute to a further understanding of the potential mechanism of quorum-sensing molecules and the search for new anti-adhesive drugs that reduce the formation of biofilms.

## Authors’ Contributions

EL and NS: Original idea for the study and carried out the design. SL: Collected the samples and data analysis. OK and AI: Drafted the manuscript. The manuscript was revised by all authors. All authors have read and approved the final manuscript.

## Data Availability

All the data generated in this study are in the published article.
